# Changes in Emotional-Behavioral Functioning Among Pre-school Children Following the Initial Stage Danish COVID-19 Lockdown and Home Confinement

**DOI:** 10.3389/fpsyg.2021.643057

**Published:** 2021-05-31

**Authors:** Ina Olmer Specht, Jeanett Friis Rohde, Ann-Kristine Nielsen, Sofus Christian Larsen, Berit Lilienthal Heitmann

**Affiliations:** ^1^The Parker Institute, Research Unit for Dietary Studies, Bispebjerg og Frederiksberg Hospital, Frederiksberg, Denmark; ^2^The Boden Institute of Obesity, Nutrition, Exercise, and Eating Disorders, University of Sydney, Sydney, NSW, Australia; ^3^The Department of Public Health, Section for General Practice, University of Copenhagen, Copenhagen, Denmark

**Keywords:** coronavirus, lockdown, child development, SDQ, child mental wellbeing

## Abstract

Unintended negative outcomes on child behavior due to lockdown and home confinement following the corona virus disease (COVID-19) pandemic needs highlighting to effectively address these issues in the current and future health crises. In this sub-study of the ODIN-study, the objectives were to determine whether the Danish lockdown and home confinement following the COVID-19 pandemic affected changes in emotional-behavioral functioning of pre-school-aged children using the validated Strength and Difficulties Questionnaire (SDQ) answered by parents shortly before lockdown and 3 weeks into lockdown, and moreover, to examine whether baseline family and social characteristics could predict change in child emotional-behavioral functioning during lockdown. Parents of 40 (82%) children with a mean(SD) age of 5.0(0.7) completed the baseline questionnaire and the lockdown follow-up questionnaire. The SDQ-Total difficulties score (SDQ-TD) and Prosocial Behavioral score (PSB) changed significantly from pre- to lockdown [SDQ-TD mean(SD): 6.0(3.8) and 7.9(5.2); *P* = 0.02, respectively and PSB mean(SD): 8.5(1.4) and 7.9(1.5); *P* = 0.03, respectively]. Attending leisure time activities before lockdown was a predicting factor of changes to the worse in the children’s SDQ-TD scores, with a mean difference in SDQ-TD between those with and without activities of 3.16 (95%CI 0.27–6.12); *P* = 0.03. In conclusion, the study showed a modest decrease in child-emotional behavioral functioning during the COVID-19 lockdown, potentially due to parental stress. Although these results might not be generalizable due to small sample size and selected population, the results point to a need of a greater awareness of child mental wellbeing during a lockdown situation.

## Introduction

A corona virus disease (COVID-19) pandemic was declared on March 11 by the World Health Organization (WHO, 2020). At 9 P.M. the same day the Danish Prime Minister announced lockdown of all non-critical functioning public institutions like kindergartens, schools, sport activities etc. Public employees with jobs that were non-essential in relation to care and treatment were sent on paid leave, and employees in the private sector were encouraged to work from home when possible. This was done to contain and mitigate spread of COVID-19 in Denmark. Although the lockdown was to be initiated starting from March 16th 2020, many parents withdrew their children from kindergartens already on March 12th. Similar strategies of lockdowns were implemented in many countries world-wide. Due to the pandemic affecting millions of peoples, drastic changes were required to deal with the COVID-19 spread, but these radical societal changes may also be accompanied by unintended adverse psychosocial outcomes (Mackolil and Mackolil, 2020).

In the Scandinavian countries, up to 97% of children at the age of 3–5 years are attending kindergartens (Social Protection in the Nordic Countries, 2014). At lockdown, the social relations were mainly limited to closest family members, and leisure time activities in sport clubs, social group activities and playgrounds were closed. Since routines are important for children at that age, the sudden isolation and the potential feeling of loss of contact with friends, kindergarten caregivers, and social activities might negatively affect the mental wellbeing of the child (Golberstein et al., 2020; Wang et al., 2020). Indeed, a study from India showed that children and adolescents at the age of 9–18 years quarantined due to the coronavirus compared to children and adolescents not quarantined, reported significantly more psychological problems, especially fear, nervousness, and annoyance (Saurabh and Ranjan, 2020). Many organizations imposed a remote work policy due to the pandemic, forcing individuals to work from home while taking care of their children due to the closing of childcares and schools. An Italian cross-sectional study showed that confinement due to COVID-19 and the changes in daily routine may adversely have affected parental stress, especially among parents with changed working routines (Cusinato et al., 2020). Parental stress has previously been found associated with family conflicts and child behavior problems (Rossman and Rosenberg, 1992). It is well known that adversities within the family dynamic like familiar conflicts and aggressions and unsupportive or neglectful relationships can have adverse consequences for the child’s mental health (Repetti et al., 2002). Thus, this parental stress and increased pressure on the family, with parents having to work from home while taking care of their children in a pandemic health crisis, and the overall social distancing, health related, and potential economic worries and reduced access to support services, might affect the children’s emotional-behavioral function (Fegert et al., 2020; Mackolil and Mackolil, 2020).

The primary aim of this study was therefore to investigate changes in emotional-behavioral functioning of kindergarten-aged children using the validated Strength and Difficulties Questionnaire (SDQ) from right before to 3 weeks into the coronavirus lockdown and home confinement. Secondly, to examine whether family and social characteristics predicted changes in child emotional-behavioral functioning as a consequence of COVID-19 lockdown.

## Methods and Research Procedure

### Participants and Research Procedures

This study was based on data from the ongoing study “ODIN, Outdoor kindergartens—the healthier choice?” The aim of the ODIN study is to investigate physical activity and sleep among kindergarten children in the Copenhagen area, Denmark. Randomly selected parents from three rotation kindergartens (kindergartens rotation from 1 week in their outdoor base and 1 week in their city base) in the Copenhagen area were handed written information about the project in the period from January 2020 to February 2020 (*n* = 100). Seventy-five agreed to participate, and 65 received an online baseline questionnaire distributed by REDCap (Harvey, 2018). The ODIN study was paused due to the coronavirus lockdown, however, parents of 49/65 healthy children had already answered the online baseline questionnaire between the 20th of February 2020 to the 11th of March 2020 in relation to demographics, family, leisure time activities, health, and the SDQ.

In start April 2020, 3 weeks into the Danish lockdown, the same parents were asked to fill in a similar questionnaire distributed through REDCap with information on SDQ referring to the lockdown period. Parents not answering the follow-up questionnaire or with missing SDQ baseline information were excluded from the analysis (*n* = 9).

### Measures

#### The Strength and Difficulties Questionnaire

The SDQ parent version for 2–4-year-old was used. SDQ has 25-items and screen for child prosocial behavior and emotional- and behavioral problems. For each question, the parents are asked to consider the child’s behavior over the past 6 months by the following answer categories: Not True, Somewhat True or Certainly True. In the lockdown follow-up questionnaire, we asked the parents to provide their answers in relation to the past 3 weeks of self-isolation.

The SDQ can be divided into five scales; “Emotional symptoms,” “Conduct problems,” “Hyperactivity,” “Peer relationship problems,” and “Prosocial behavior (PSB),” with scores ranging from 0 to 10 points where higher scores on the problem subscales indicate more emotional-behavioral difficulties, and lower scores on the prosocial subscale indicate lower prosocial behaviors. The scores within all categories except PSB were summed to a Total Difficulties score (SDQ-TD) ranging 0–40 points, with 0–13 points defined as normal SDQ-TD (Strengths and Difficulties Questionnaire, 2012). The score from the PSB scale was not incorporated into the SDQ-TD score, as absence of pro-social behaviors differs conceptually from the presence of psychological difficulties (Goodman, 1997). The PSB score was hence used as a variable by itself. An externalizing score can be obtained by summing the conduct and hyperactivity scales. Also, an internalizing score can be obtained by summing the emotional and peer problems scales. Both scores range from 0 to 20 points, with a higher score referring to more externalizing or internalizing behavior, respectively. The questionnaire has been validated in a Danish content (Obel et al., 2004).

#### Demographic Variables

We investigated potential family and social predictors of changes in SDQ measures during lockdown. These predictors were sex (female/male), child age (3.5–5.0/5.1–6.8 years), siblings (yes/no), sibling order (youngest/oldest), parent rated child health pre-lockdown (better/the same as peers), child leisure time activities pre-lockdown (yes/no), parent leisure time activities pre-lockdown (yes/no), joint leisure time activities child and parent together (yes/no), and information about which parent filed in the questionnaire (mother/father). Parent rated child health pre-lockdown was defined according to the question: How is your child’s health as compared to peers? With the opportunity to answer “Better,” The same as peers’ or “Worse.” Since only two children were rated as “worse,” these children were deleted in the analysis of parent rated child health and the variable was dichotomized to “better” or “the same as peers.” Child leisure time activities were defined according to the following two questions; “Does the child attend sport activities outside kindergarten hours?” and “Does the child attend other leisure time activities outside kindergarten hours not categorized as sports?” Similar questions were posed to the parents about their leisure time activities. The question defining leisure time activities that both parents and children took part in was “Does the family have joint leisure activities?”

Demographic variables not included as predicting variables were child ethnicity (Western origin/not Western origin), parental education (less than 3 years of higher education/bachelor’s degree/master’s degree) and whether parents were living together (yes/no). These variables were not included as potential predictions in change of SDQ since the variance within each variable was too small.

### Ethical Approval

All procedures performed in studies involving human participants were in accordance with the ethical standards of the institutional and/or national research committee and with the 1964 Helsinki declaration and its later amendments or comparable ethical standards. Permission from the Capital Region Data Agency was granted (Journal no.: P-2020-54). Informed consent was obtained from all individual participants included in the study both at baseline and follow-up.

### Statistical Analysis

Descriptive statistics were provided as percentages or means and standard deviation (SD). A paired *t*-test was used to evaluate changes in SDQ scores from pre-lockdown (baseline) to lockdown (follow-up). General linear models were used to analyze potential predictors affecting changes in pre-lockdown to lockdown, with the outcome variable being the difference between pre-lockdown and lockdown SDQ-TD or PSB scores, and the exposure being the potential predictor adjusted for the baseline level of the SDQ-TD or the -PSB score. The predicting factors were sex (female/male), child age (3.5–5.0/5.1–6.8 years), siblings (yes/no), sibling order (youngest/oldest), child leisure time activities pre-lockdown (yes/no), parent leisure time activities pre-lockdown (yes/no), joint leisure time activities child and parent together (yes/no), parent rated child health pre-lockdown (better/the same as peers), and information about which parent filed in the questionnaire (mother/father).

Normality of continuous variables and model assumptions were assessed.

All statistical analyses were performed using SAS 9.4. Statistical significance was declared if a two-sampled *p*-value was less than 0.05.

## Results

Forty (82%) out of 49 completed the lockdown follow-up questionnaire. Among the nine parents not answering the follow-up questionnaire, only five had completed the baseline SDQ. The baseline SDQ scores were significantly lower among the 40 answering the follow-up than the five only answering the baseline questionnaire (*p*-value = 0.02).

Overall, parents were highly educated: 61% having a master’s degree and 34% a bachelor’s degree, 95.1% were employed and had a Westerns origin, and 92.7% of the parents were living together. The child mean (SD) age was 5.0 years (0.7).

Almost all (97.6%) of the children were within the normal SDQ-TD scores of 0–13 points at pre-lockdown and during lockdown (92.7%).

When investigating the changes in SDQ scores, the primary aim, the SDQ-TD scores changed significantly from pre- to lockdown with a higher (worse) score during lockdown [SDQ-TD mean (SD): 6.0 (3.8) and 7.9 (5.2); *P* = 0.02, respectively, Table 1]. Similar tendencies were observed among the results from the five scales, significantly so for Hyperactivity and the PSB scores (Table 1). Also, the children were rated more externalizing during lockdown (Table 1).

**TABLE 1:** Mean and SD in SDQ domains from pre-lockdown and lockdown, *N* = 40.

	**Mean**	**SD**	***P*-value^*a*^**
SDQ-TD score			0.02
Pre-lockdown	6.0	3.8	
Lockdown	7.9	5.2	
Emotional problems score			0.69
Pre-lockdown	1.5	1.5	
Lockdown	1.6	1.6	
Conduct problems score			0.08
Pre-lockdown	1.6	1.5	
Lockdown	2.1	1.8	
Hyperactivity score			0.01
Pre-lockdown	2.5	2.4	
Lockdown	3.6	2.9	
Peer problem score			0.15
Pre-lockdown	0.4	0.7	
Lockdown	0.7	1.2	
PSB score^b^			0.03
Pre-lockdown	8.5	1.4	
Lockdown	7.9	1.5	
Externalizing^c^			0.006
Pre-lockdown	4.1	3.1	
Lockdown	5.6	4.0	
Internalizing^c^			0.34
Pre-lockdown	1.9	1.8	
Lockdown	2.3	2.3	

*^*a*^Paired *t*-test. ^*b*^PSB, Prosocial behavior. ^*c*^Externalizing or Internalizing scores were done by summing the conduct and hyperactivity scales or the emotional and peer problems scales, respectively, ranging from 0 to 20 points.*

When investigating whether family and social characteristics predicted changes in child SDQ, the secondary aim, attending leisure time activities before lockdown predicted changes in SDQ-TD to the worse, with a mean difference in SDQ-TD scores between those with and those without leisure times activities of 3.16 (95% CI: 0.27;6.12) (Table 2). Increased SDQ scores were also revealed among children without siblings; however, the sample size was small with only four children without siblings. There was a tendency toward increased SDQ-TD and decreased PSB scores among girls, however, differences were non-significant between sexes. Also, parents with leisure time activities before lockdown scores their children’s SDQ-TD and PSB worse, but not significantly between parents with or without leisure time activities (Table 2).

**TABLE 2:** Changes in SDQ-TD or PSB scores from pre- to lockdown stratified by predicting factors. The difference (Diff.) corresponds to the mean difference between the dichotomized predicting factor.

	***n***	**SDQ-TD Change^*a*^**	**95% CI**		***p*-diff.**	**PSB Change^*a*^**	**95% CI**		***p*-diff.**
Female	22	**2.25**	**0.22**	**4.27**		**−0.70**	**−1.34**	**−0.05**	
Male	18	1.25	–0.99	3.49		–0.51	–1.20	0.19	
Diff.	40	0.99	–2.08	4.06	0.29	–0.19	–1.14	0.76	0.69
Age 3.5–5 years	17	2.35	–0.13	4.83		**−0.95**	**−1.68**	**−0.21**	
Age 5.1–6.8 years	15	0.83	–1.52	3.96		–0.66	–1.44	0.12	
Diff.	32	1.13	–2.62	4.88	0.54	–0.28	–1.36	0.79	0.59
Siblings	36	1.29	–0.18	2.75		–0.52	–1.01	0.03	
No siblings	4	**6.41**	**1.99**	**10.82**		–1.46	–2.95	0.04	
Diff.	40	**−5.12**	**−9.77**	−**0.46**	**0**.**03**	0.94	–0.63	2.52	0.23
Youngest sibling	21	**1.63**	**0.38**	**2.89**		–0.41	–0.98	0.17	
Oldest sibling	14	1.34	–0.20	2.88		–0.51	–1.23	0.22	
Diff.	35	0.12	–1.98	2.22	0.91	0.10	–0.83	1.03	0.83
Leisure time activities	26	**2.92**	**1.19**	**4.64**		**−0.69**	**−1.27**	−**0.10**	
No leisure time activities	14	–0.27	–2.63	2.08		–0.46	–1.27	0.34	
Diff.	40	**3.19**	**0.27**	**6.12**	**0**.**03**	–0.22	–1.22	0.77	0.65
Children with parent-rated health as better than peers	13	2.27	–0.37	4.91		–0.44	–1.28	0.39	
Children with parent-rated the same as peers	25	1.66	–0.24	3.56		**−0.71**	**−1.33**	**−0.09**	
Diff.	38	0.62	–2.64	3.87	0.70	0.27	–0.79	1.33	0.61
Parental leisure activities	25	**2.25**	**0.36**	**4.15**		**−0.66**	**−1.27**	**−0.05**	
No parental leisure activities	16	1.12	–1.21	3.45		–0.53	–1.29	0.23	
Diff.	40	1.13	–1.87	4.14	0.45	–0.13	–0.10	0.84	0.79
Joint leisure time activities child and parents together	8	2.80	–0.55	6.15		–0.68	–1.75	0.39	
No child and parent cooperating leisure time activities	33	1.55	–0.10	3.20		−**0.59**	−**1.12**	−**0.07**	
Diff.	40	–1.25	–5.01	2.52	0.51	0.59	–1.11	1.28	0.88
Mothers filed out the questionnaire	32	**2.29**	**0.67**	**3.91**		**−0.63**	**−1.16**	**−0.11**	
Fathers filed out the questionnaire	8	–0.16	–3.43	3.11		–0.50	–1.58	0.56	
Diff.	40	2.45	–1.22	6.11	0.18	–0.13	–1.32	1.07	0.83

*^*a*^Adjusted for baseline SDQ-TD or prosocial score, respectively. Diff. is corresponding to the difference between the dichotomized predicting factor, whereas the estimated of the dichotomized predicting factors are the difference from baseline to follow-up. Statistical significant associations are bolded.*

## Discussion

Despite a small sample size, the current study indicated that lockdown may have had adverse consequences for child emotional-behavioral function in relation to the home confinement during the COVID-19 outbreak as hypothesized by others (Golberstein et al., 2020; Prime et al., 2020; Wang et al., 2020). Our results from this pretest-posttest study design showed that after 3 weeks of home confinement the SDQ-TD increased, which seemed driven by the hyperactivity score, also confirmed by an increase in the externalizing behavior score. However, the scores were within the normal SDQ range. An Italian cross-sectional study undertaken approximately 50 days within the COVID-19 confinement did not report an increased child SDQ-TD score when comparing answers from 463 parents with an normative Italian population (Cusinato et al., 2020). However, they did observe a significant increase in the hyperactivity score among confined children, as also found by others (Di Giorgio et al., 2020). The first 3 weeks of lockdown might have been a particularly difficult period for the family, where they needed to adjust to the new situation, facing unprecedented increase in anxiety and stressors (Repetti et al., 2002; Prime et al., 2020). In total, 95.1% of the parent were employed, and since the majority had high education they might have worked from home. The questionnaires were filled in by parents, and changes in their work-life balance and social activities could have affected scoring of the children’s SDQ during lockdown, since SDQ has been shown to predict parental stress during COVID-19 (Cusinato et al., 2020). Thus, the results might reflect parental stress due to the home confinement situation rather than emotional-behavioral function of their child. The results showing increasing child SDQ-TD scores among parents with leisure time activities before lockdown might confirm this. Previous studies did however, not show that changes in working routines affected the children’s wellbeing (Cusinato et al., 2020; Di Giorgio et al., 2020). We did not have information on parental mental health which could have changed prior to and post lockdown, and thus affected the SDQ scoring. Parents may also have been more aware of their children’s behavior during lockdown than pre-lockdown, and thus the lockdown SDQ scores might report the true emotional-behavioral function better than pre-lockdown. However, since we found an increase in externalizing behavior particularly among specific groups of children, e.g., children with leisure time activities, our result might be valid. The increase in externalizing behavior could be due to loss of private space, room for active play and socializing with peers, but also a feeling of a greater change in routines. Before lockdown, children with leisure time activities spent less time home than children without leisure time activities, and thus potentially less time with the parents. However, since the children in the current study are young, activities often include the presence of a parent.

A study of 41 children with obesity showed unfavorable changes in their eating, activity, and sleep behaviors during COVID-19 lockdown as compared to before lockdown (Pietrobelli et al., 2020). Also, a Canadian cross-sectional study of 1,472 children and youth reported lower parental reported physical activity and more sedentary behavior during COVID-19 lockdown (Moore et al., 2020). These results support our findings since disruption in the biological rhythm may cause emotional and behavioral changes (Reyes et al., 2019).

The major advantage of the current study was that we had SDQ measurements taken right before as well as during COVID-19 lockdown within the same children. A previous study among 245 mothers and their kindergarten-aged children showed similar results in relation to increased SDQ scores after 3 weeks of confinement, however, they asked the participants to fill in the survey in relation to their present situation during the quarantine and retrospectively thinking of the week before COVID-19 lockdown (Di Giorgio et al., 2020).

However, our study has some limitations. The sample size was small; however, despite this we were still able to show significant changes in SDQ. The small sample size made it impossible to make important interaction analysis across the sample. Also, due to both the small sample size and the exploratory nature of these analyses, we did not adjust for multiple testing, and thus the results should be interpreted with caution due to the risk of type I errors. Had Bonferroni adjustment been applied to account for the number of tests conducted, many of the findings would not have been statistically significant. The SDQ questionnaire is validated as an questionnaire investigating child strength and difficulties the past 6 months (Goodman, 1997) and the short follow-up of only 3 weeks is a potential limitation, however, we were interested in investigating the SDQ during and not after lockdown. Additionally, it is possible that the parents remembered their previous answers at baseline which could have affected their scoring. However, despite this, they did report significant change. We decided to not exclude the PSB scores, which are related to the relations to the child’s peers, generated by the SDQ questionnaire, although the children were isolated from peers from the kindergarten, the children might have had contact to other children like family members or few close friends.

### Conclusion

The present study suggests a tendency toward adverse consequences on child emotional-behavioral function in relation to the home confinement during the COVID-19 outbreak, which has also been suggested by others. However, the results of the present study might not be generalizable to other pre-school aged children as children in the present study were from better educated families with a better job security than the general Danish population. Also, the results might not be generalizable to children not attending kindergartens, or non-rotating kindergartens. Our results point to a need to increase awareness of child emotional-behavioral function during a lockdown situation, like potential future waves of COVID-19. This could include more video contact from kindergarten teachers, encouragement to participate in web-based initiatives, or online psychological counseling (Wang et al., 2020). The collateral effects of the COVID-19 pandemic are not only related to physical illness, but extend to the much broader population, potentially with long-term consequences.

## Data Availability Statement

The raw data supporting the conclusions of this article will be made available by the authors, without undue reservation, when approval by the Danish Capital Region Data Agency has been granted.

## Ethics Statement

Ethical review and approval was not required for the study on human participants in accordance with the local legislation and institutional requirements. Written informed consent to participate in this study was provided by the participants’ legal guardian/next of kin.

## Author Contributions

IS and SL conducted the analytical plan. IS analyzed the data and drafted the manuscript with help from all authors. IS had full access to all the data in the study and takes responsibility for the integrity of the data and the accuracy of the data analysis. All authors designed the study and approved the final manuscript for submission.

## Conflict of Interest

The authors declare that the research was conducted in the absence of any commercial or financial relationships that could be construed as a potential conflict of interest.
